# Striking antibody evasion of SARS-CoV-2 Omicron sub-lineages BQ.1.1, XBB.1 and CH.1.1

**DOI:** 10.1093/nsr/nwad148

**Published:** 2023-05-23

**Authors:** Bin Ju, Huimin Guo, Miao Wang, Qing Fan, Senlin Shen, Xuejiao Liao, Jie Jiang, Haiyan Wang, Fuxiang Wang, Zheng Zhang

**Affiliations:** Institute for Hepatology, National Clinical Research Center for Infectious Disease, Shenzhen Third People's Hospital, and The Second Affiliated Hospital, School of Medicine, Southern University of Science and Technology, China; Guangdong Key Laboratory for Anti-Infection Drug Quality Evaluation, China; Institute for Hepatology, National Clinical Research Center for Infectious Disease, Shenzhen Third People's Hospital, and The Second Affiliated Hospital, School of Medicine, Southern University of Science and Technology, China; Institute for Hepatology, National Clinical Research Center for Infectious Disease, Shenzhen Third People's Hospital, and The Second Affiliated Hospital, School of Medicine, Southern University of Science and Technology, China; Institute for Hepatology, National Clinical Research Center for Infectious Disease, Shenzhen Third People's Hospital, and The Second Affiliated Hospital, School of Medicine, Southern University of Science and Technology, China; Institute for Hepatology, National Clinical Research Center for Infectious Disease, Shenzhen Third People's Hospital, and The Second Affiliated Hospital, School of Medicine, Southern University of Science and Technology, China; Institute for Hepatology, National Clinical Research Center for Infectious Disease, Shenzhen Third People's Hospital, and The Second Affiliated Hospital, School of Medicine, Southern University of Science and Technology, China; Institute for Hepatology, National Clinical Research Center for Infectious Disease, Shenzhen Third People's Hospital, and The Second Affiliated Hospital, School of Medicine, Southern University of Science and Technology, China; Institute for Hepatology, National Clinical Research Center for Infectious Disease, Shenzhen Third People's Hospital, and The Second Affiliated Hospital, School of Medicine, Southern University of Science and Technology, China; Department of Infectious Diseases, National Clinical Research Center for Infectious Disease, Shenzhen Third People's Hospital, and The Second Affiliated Hospital, School of Medicine, Southern University of Science and Technology, China; Institute for Hepatology, National Clinical Research Center for Infectious Disease, Shenzhen Third People's Hospital, and The Second Affiliated Hospital, School of Medicine, Southern University of Science and Technology, China; Guangdong Key Laboratory for Anti-Infection Drug Quality Evaluation, China; Shenzhen Research Center for Communicable Disease Diagnosis and Treatment of the Chinese Academy of Medical Sciences, China

The recent emergence of various B.1.1.529 (Omicron) sub-lineages of severe acute respiratory syndrome coronavirus 2 (SARS-CoV-2) poses an enormous challenge for the prevention and treatment of coronavirus disease 2019 (COVID-19) [1,2]. BA.2 and BA.5 sub-lineages, with marked antibody evasion, have been dominant in many countries and regions worldwide [3,4]. Recently, a series of BA.2- and BA.5-related progeny and recombinant variants have been isolated and identified, such as BJ.1, BA.2.75, BA.2.3.20, BQ.1.1, XBB.1 and CH.1.1 [5], whose ability to escape immune plasma and neutralizing antibodies (nAbs) needs to be evaluated immediately.

As shown in Fig. 1A and Supplementary Fig. S1, BJ.1, BA.2.75 and BA.2.3.20 were three progeny sub-variants of BA.2, carrying 15, 9 and 10 alterations, respectively [5]. BQ.1.1 was the progeny virus of BA.5, with three additional mutations. CH.1.1 was one of the most frequently observed BA.2.75 sub-variants, carrying additional R346T, K444T, L452R and F486S mutations [6]. XBB was a recombinant variant between BJ.1 and BA.2.75, which further evolved to XBB.1 with an additional G252V substitution. Here, we construct SARS-CoV-2 pseudoviruses of BJ.1, BA.2.75, BA.2.3.20, BQ.1.1, XBB.1 and CH.1.1, and measure their capacity to evade antibodies of the plasma of BA.4 or BA.5 breakthrough infected donors and monoclonal nAbs in a head-to-head comparison with the wild type (WT), BA.2 and BA.4/5 (BA.4 and BA.5 sharing the same amino acid sequence in the spike region).

**Figure 1.**
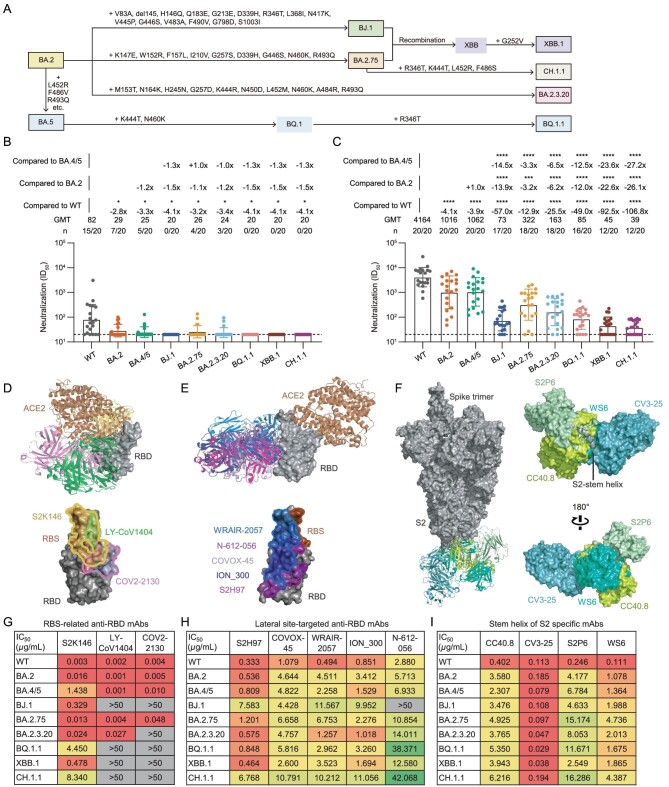
Neutralization resistance of SARS-CoV-2 Omicron sub-lineages to BA.4 or BA.5-infected human plasma and existing monoclonal bnAbs. (A) A schematic diagram of the evolution of several SARS-CoV-2 Omicron sub-lineages. Some additional mutations acquired by BJ.1, BA.2.75, BA.2.3.20, BQ.1.1, XBB.1 and CH.1.1 are displayed. (B) and (C) The neutralization of 20 BA.4 or BA.5 breakthrough infected human plasma samples collected in the early stage of infection (B: Visit 1) and in another follow-up (C: Visit 2, an interval of 7–15 days) against WT SARS-CoV-2, BA.2, BA.4/5, BJ.1, BA.2.75, BA.2.3.20, BQ.1.1, XBB.1 and CH.1.1, respectively. ID_50_ values are means of two independent experiments. Data are presented as geometric mean values ± standard deviation (SD). The number, GMT, fold change and significance-of-difference are labeled at the top. ‘−’ represents a decreased value and ‘+’ an increased value. The statistical significance was performed using the two-tailed Kruskal-Wallis test with paired Wilcoxon's multiple-comparison test. ****, *P* < 0.0001; ***, *P* < 0.001; *, *P* < 0.05. (D)–(F) Overall structure and footprint of bnAbs in complex with SARS-CoV-2. S2K146, LY-CoV1404 and COV2-2130 recognize the epitopes overlapping with the RBS (D). S2H97, COVOX-45, WRAIR-2057, ION_300 and N-612-056 target the lateral RBD site (E). CC40.8, CV3-25, S2P6 and WS6 bind to the stem helix in S2 of the spike (F). (G)–(I) The neutralization of monoclonal bnAbs against WT SARS-CoV-2, BA.2, BA.4/5, BJ.1, BA.2.75, BA.2.3.20, BQ.1.1, XBB.1 and CH.1.1. IC_50_ values are means of two independent experiments. The neutralization potency is marked in a different color. Red: high, yellow: moderate, green: weak, gray: non-neutralization (IC_50_ > 50 μg/mL).

The plasma samples were obtained from 20 BA.4- or BA.5-infected individuals who had received at least two doses of SARS-CoV-2 vaccines based on the WT virus or WT spike sequence, including adenovirus-vectored, inactivated, mRNA and recombinant protein vaccines (Supplementary Fig. S2, Tables S1 and S2). In the early stage of breakthrough infection (Visit 1, 0–5 days post positive PCR test), plasma nAbs were detectable in most individuals (15/20), with geometric mean titer (GMT) of 82 against the vaccine stimulation-related WT SARS-CoV-2 pseudovirus. By contrast, most plasma samples lost their neutralizing activities against BA.2, BA.4/5, BJ.1, BA.2.75, BA.2.3.20, BQ.1.1, XBB.1 and CH.1.1 (Fig. 1B and Supplementary Fig. S3). In another follow-up (Visit 2, 7–15 days after Visit 1), the overall GMTs of plasma nAbs against all tested variants had increased to some extent (Fig. 1C and Supplementary Fig. S4), due to the BA.4 or BA.5 breakthrough infection. However, these plasma samples still showed 4.1-, 3.9-, 57.0-, 12.9-, 25.5-, 49.0-, 92.5- and 106.8-fold reduction in the GMT against BA.2, BA.4/5, BJ.1, BA.2.75, BA.2.3.20, BQ.1.1, XBB.1 and CH.1.1, respectively, as compared with that against WT. BA.2 and BA.4/5 were previous Omicron sub-lineages, having relatively higher homology with WT virus than BJ.1, BA.2.75, BA.2.3.20, BQ.1.1, XBB.1 and CH.1.1. Therefore, the GMTs of the nAbs of these plasma samples against WT, BA.2 and BA.4/5 were significantly higher than against other variants (Fig. 1C). CH.1.1 displayed the largest degree of neutralizing antibody evasion induced by the BA.4 or BA.5 breakthrough infection among all tested Omicron sub-lineages.

Meanwhile, these paired plasma samples between Visit 1 and Visit 2 provided an opportunity to quantify the enhancement of the BA.4 or BA.5 breakthrough infection in WT-vaccinated people. A similar analysis was performed to evaluate the BA.2 breakthrough infection in our previous study [7]. As shown in Supplementary Fig. S5, the fold change (FC) of GMTs against WT was the highest (51.0-fold), followed by BA.4/5, BA.2, BA.2.75, BA.2.3.20, BQ.1.1, BJ.1, XBB.1 and CH.1.1. Of note, it was difficult for the BA.4 or BA.5 breakthrough infection to promote neutralization against CH.1.1. Overall, due to the original antigenic sin (or so-called immune imprinting) of the initial WT vaccination, these plasma samples from BA.4 or BA.5 breakthrough infected individuals acquired weaker neutralization against subsequent Omicron sub-lineages, such as BQ.1.1, XBB.1 and CH.1.1.

In this study, we also evaluated the neutralization resistances of these recently emerged Omicron sub-lineages to eight previous monoclonal broad nAbs (bnAbs) directed to the receptor-binding domain (RBD) of the spike and four S2-specific bnAbs (Supplementary Fig. S6). According to their well-defined structural information, we analyzed their binding epitopes and broadly neutralizing activities in parallel. Among these anti-RBD bnAbs, S2K146, LY-CoV1404 and COV2-2130 recognize the epitopes overlapping with the receptor-binding site (RBS), displaying both good broad-spectrum and potent neutralizing capacity (Fig. 1D). In contrast, another five anti-RBD bnAbs (S2H97, COVOX-45, WRAIR-2057, ION_300 and N-612-056) with moderate potencies target the lateral site, barely overlapping with the RBS (Fig. 1E). Except for the RBD, so far, another main target for bnAbs is the S2 domain of the spike. For example, CC40.8, CV3-25, S2P6 and WS6 bind to the stem helix and disturb further conformational change (Fig. 1F).

Although LY-CoV1404 and COV2-2130 could potently neutralize WT SARS-CoV-2, BA.2, BA.4/5 and BA.2.75, they totally lost their neutralization against BJ.1, BQ.1.1, XBB.1 and CH.1.1. Meanwhile, the neutralization of COV2-2130 was impaired against BA.2.3.20. S2K146 still maintained overall neutralizing activities against all tested variants, despite marked reduction of potencies against BA.4/5, BQ.1.1 and CH.1.1 (Fig. 1G). In contrast, the majority of the lateral site-targeted bnAbs (4/5) and all of the tested S2 bnAbs targeting the stem helix (4/4) could neutralize recently emerged Omicron sub-lineages including BJ.1, BA.2.75, BA.2.3.20, BQ.1.1, XBB.1 and CH.1.1, albeit exhibiting moderate or weak potencies against some particular variants (Fig. 1H and I).

In summary, our data showed that BJ.1, BA.2.75, BA.2.3.20, BQ.1.1, XBB.1 and CH.1.1 exhibited large degrees of enhanced antibody evasion from BA.4 or BA.5 breakthrough infected human plasma samples and existing monoclonal bnAbs. Despite the different kinds of vaccines and different doses of immunizations that were involved in this study, all vaccines used here were derived from the WT virus or designed based on the WT spike sequence. From this perspective, this cohort could be used to analyze the influence of the WT-vaccination immune background on the boosting effect of BA.4/5 breakthrough infection. The original antigenic sin phenomenon was also observed in the BA.4/5 breakthrough infection, similar to that previously reported in the BA.1 and BA.2 breakthrough infections [5,8]. Consistent with previous studies [5,6,9], a wide range of Omicron sub-lineages escaped LY-CoV1404 (an approved antibody drug, also known as bebtelovimab) for the first time. LY-CoV1404 had always been regarded as the best bnAb before the BJ.1, BQ.1.1, XBB.1 and CH.1.1 variants emerged [2,10]. However, our study highlights that some bnAbs binding to the lateral RBD epitope and the S2 stem helix could provide a necessary supplement for the development of broad antibody drug candidates in order to tackle the ongoing COVID-19 pandemic.

## DATA AVAILABILITY

We are happy to share reagents and information upon request.

## Supplementary Material

nwad148_Supplemental_File
