# Supplementary material for: Striking antibody evasion of SARS-CoV-2 Omicron sub-lineages BQ.1.1, XBB.1 and CH.1.1
Source: Natl Sci Rev. 2023 May 23;10(8):nwad148. doi: 10.1093/nsr/nwad148 (PMC10309178; doi:10.1093/nsr/nwad148)
Supplement: nwad148_Supplemental_File [file nwad148_supplemental_file.pdf]

Supplementary information

Materials and Methods

Study approval and plasma samples

This study was approved by the Ethics Committee of Shenzhen Third People's Hospital, China (approval number: 2021-030). All participants had provided written informed consent for sample collection and subsequent analysis. The plasma samples were collected from 20 individuals infected with BA.4 or BA.5 variants, all of which had received at least two doses of vaccines designed on the wild-type (WT) SARS-CoV-2. All plasma samples were stored at -80°C in the Biobank of Shenzhen Third People's Hospital and heat-inactivated at 56°C for 1 h before use.

Structural analysis of SARS-CoV-2 RBD-specific and anti-S2 monoclonal antibodies (mAbs)

All structure data were downloaded from the protein data bank (PDB) and PDB codes were provided: S2K146 (7TAS), LY-CoV1404 (7MMO), COV2-2130 (7L7E), S2H97 (7M7W), COVOX-45 (7PRY), WRAIR-2057 (7N4I), ION_300 (7BNV), N-612-056 (7S0B), CC40.8 (7SJS), CV3-25 (7RAQ), S2P6 (7RNJ), WS6 (7TCQ), ACE2 (6M0J), and spike trimer (7DWZ). The structural models were made using PyMOL Molecular Graphics System 2.5.4. The potential interactions were calculated by PISA v1.52 (https://www.ebi.ac.uk/pdbe/pisa/).

Expression and purification of mAbs

All gene sequences of tested mAbs were downloaded from the National Center of Biotechnology Information (NCBI) and the PDB, which were synthesized and cloned into full-length IgG1 expression vectors (Sangon Biotech or GenScript). Paired heavy and light chains were co-transfected into the 293 F cells. Using Protein-A columns, mAbs were purified after a five-day culture according to the manufacturer's instructions (Senhui Microsphere Technology), as previously described [1-3].

Generation of SARS-CoV-2 pseudoviruses

The spike genes of WT SARS-CoV-2 and Omicron variants (BA.2, BA.4/5, BJ.1, BA.2.75, BA.2.3.20, BQ.1.1, XBB.1, and CH.1.1) were synthesized (GenScript) and then inserted into the pVAX1 vector. The pseudovirus was generated by co-transfection with each spike plasmid and HIV-1 env-deficient backbone vector (pNL4-3.Luc.R-E-) into the HEK-293T cells, as previously described [4, 5]. After 2-day co-transfection, all culture supernatants were harvested, clarified by centrifugation, and then stored at -80°C. Optimal infectious titer was determined by measuring the luciferase activity in the HEK-293T-hACE2 cells using Bright-Lite Luciferase reagent (Vazyme Biotech).

Detailed sequence information of spike protein used in this study was listed below, respectively.

Wild-type (WT) SARS-CoV-2: accession number: NC_045512;

SARS-CoV-2 BA.2: accession number: EPI_ISL_9652748:

T19I, L24S, del25/27, G142D, V213G, G339D, S371F, S373P, S375F, T376A, D405N, R408S, K417N, N440K, S477N, T478K, E484A, Q493R, Q498R, N501Y, Y505H, D614G, H655Y, N679K, P681H, N764K, D796Y, Q954H, N969K;

SARS-CoV-2 BA.4/5: accession number: EPI_ISL_11542550:

T19I, L24S, del25/27, del69/70, G142D, V213G, G339D, S371F, S373P, S375F, T376A, D405N, R408S, K417N, N440K, L452R, S477N, T478K, E484A, F486V, Q498R, N501Y, Y505H, D614G, H655Y, N679K, P681H, N764K, D796Y, Q954H, N969K;

BA.4 and BA.5 shared the same amino acid sequence of spike protein, so we used BA.4/5 to represent the sequences of BA.4 and BA.5 in this study.

SARS-CoV-2 BJ.1: accession number: EPI_ISL_14167044:

T19I, L24S, del25/27, V83A, G142D, del145, H146Q, Q183E, V213E, G339H, R346T, L368I, S371F, S373P, S375F, T376A, D405N, R408S, N440K, V445P, G446S, S477N, T478K, V483A, E484A, F490V, Q493R, Q498R, N501Y, Y505H, D614G, H655Y, N679K, P681H, N764K, D796Y, G798D, Q954H, N969K, S1003I;

SARS-CoV-2 BA.2.75: accession number: EPI_ISL_13471039:

T19I, L24S, del25/27, G142D, K147E, W152R, F157L, I210V, V213G, G257S,

G339H, S371F, S373P, S375F, T376A, D405N, R408S, K417N, N440K, G446S, N460K, S477N, T478K, E484A, Q498R, N501Y, Y505H, D614G, H655Y, N679K, P681H, N764K, D796Y, Q954H, N969K;

SARS-CoV-2 BA.2.3.20: accession number: EPI_ISL_14809314:

T19I, L24S, del25/27, G142D, M153T, N164K, V213G, H245N, G257D, G339D, S371F, S373P, S375F, T376A, D405N, R408S, K417N, N440K, K444R, N450D, L452M, N460K, S477N, T478K, E484R, Q498R, N501Y, Y505H, D614G, H655Y, N679K, P681H, N764K, D796Y, Q954H, N969K;

SARS-CoV-2 BQ.1.1: accession number: EPI_ISL_14818139:

T19I, L24S, del25/27, del69/70, G142D, V213G, G339D, R346T, S371F, S373P, S375F, T376A, D405N, R408S, K417N, N440K, K444T, L452R, N460K, S477N, T478K, E484A, F486V, Q498R, N501Y, Y505H, D614G, H655Y, N679K, P681H, N764K, D796Y, Q954H, N969K;

SARS-CoV-2 XBB.1: accession number: EPI_ISL_14917761:

T19I, L24S, del25/27, V83A, G142D, del145, H146Q, Q183E, V213E, G252V, G339H, R346T, L368I, S371F, S373P, S375F, T376A, D405N, R408S, K417N, N440K, V445P, G446S, N460K, S477N, T478K, E484A, F486S, F490S, Q498R, N501Y, Y505H, D614G, H655Y, N679K, P681H, N764K, D796Y, Q954H, N969K;

SARS-CoV-2 CH.1.1: accession number: EPI_ISL_15713635:

T19I, L24S, del25/27, G142D, K147E, W152R, F157L, I210V, V213G, G257S, G339H, R346T, S371F, S373P, S375F, T376A, D405N, R408S, K417N, N440K, K444T, G446S, L452R, N460K, S477N, T478K, E484A, F486S, Q498R, N501Y, Y505H, D614G, H655Y, N679K, P681H, N764K, D796Y, Q954H, N969K.

SARS-CoV-2 pseudovirus-based neutralization assay

To measure the neutralizing activities, plasma samples or mAbs were serially diluted. WT or variant pseudoviruses were mixed with diluted plasma or mAbs and incubated at 37°C for 1 h. Pseudovirus without plasma or mAbs was used as the virus control. The mixture was added into the HEK-293T-hACE2 cells in 96-well cell plates and incubated for 48 h at 37°C. The culture supernatant was removed and 100 μL of Bright-Lite Luciferase reagent (Vazyme Biotech) was added. After shaking at room temperature for 3 mins, the luciferase activity was

measured using Varioskan LUX multimode microplate reader (Thermo Fisher Scientific). The inhibition was determined by comparing with the virus control. The value of 50% inhibitory dilution (ID_{50}) or 50% inhibitory concentration (IC_{50}) was calculated using the Graphpad Prism 9 by log (inhibitor) vs. normalized response – Variable slope (four parameters) model. The cut-off value of neutralization was set as 1:20 dilution for plasma or 50 μg/mL for mAbs.

References:

1. Ju B, Zheng Q and Guo H *et al. Cell Res* 2022; **32**: 491-4.
2. Guo H, Jiang J and Shen S *et al. iScience* 2023; **26**: 106283.
3. Guo H, Fan Q and Song S *et al. J Clin Virol* 2022; **150-151**: 105162.
4. Ju B, Zhang Q and Ge J *et al. Nature* 2020; **584**: 115-9.
5. Ju B, Zhang Q and Wang Z *et al. Nat Immunol* 2023; **24**: 690-9.

	NTD																					
	19	24	25	26	27	69	70	83	142	145	146	147	152	153	157	164	183	210	213	245	252	257
WT	T	L	P	P	A	H	V	V	G	Y	H	K	W	M	F	N	Q	I	V	H	G	G
BA.2	I	S							D										G			
BA.2.3.20	I	S							D					T		K			G	N		D
BA.2.75	I	S							D			E	R		L			V	G			S
CH.1.1	I	S							D			E	R		L			V	G			S
BJ.1	I	S						A	D		Q						E		E			
XBB.1	I	S						A	D		Q						E		E		V	
BA.4/5	I	S							D										G			
BQ.1.1	I	S							D										G			

	RBD																										
	339	346	368	371	373	375	376	405	408	417	440	444	445	446	450	452	460	477	478	483	484	486	490	493	498	501	505
WT	G	R	L	S	S	S	T	D	R	K	N	K	V	G	N	L	N	S	T	V	E	F	F	Q	Q	N	Y
BA.2	D			F	P	F	A	N	S	N	K							N	K		A			R	R	Y	H
BA.2.3.20	D			F	P	F	A	N	S	N	K	R			D	M	K	N	K		R				R	Y	H
BA.2.75	H			F	P	F	A	N	S	N	K			S			K	N	K		A				R	Y	H
CH.1.1	H	T		F	P	F	A	N	S	N	K	T		S		R	K	N	K		A	S			R	Y	H
BJ.1	H	T	I	F	P	F	A	N	S		K		P	S				N	K	A	A		V	R	R	Y	H
XBB.1	H	T	I	F	P	F	A	N	S	N	K		P	S			K	N	K		A	S	S		R	Y	H
BA.4/5	D			F	P	F	A	N	S	N	K					R		N	K		A	V			R	Y	H
BQ.1.1	D	T		F	P	F	A	N	S	N	K	T				R	K	N	K		A	V			R	Y	H

					S2					
	614	655	679	681	764	796	798	954	969	1003
WT	D	H	N	P	N	D	G	Q	N	S
BA.2	G	Y	K	H	K	Y		H	K	
BA.2.3.20	G	Y	K	H	K	Y		H	K	
BA.2.75	G	Y	K	H	K	Y		H	K	
CH.1.1	G	Y	K	H	K	Y		H	K	
BJ.1	G	Y	K	H	K	Y	D	H	K	I
XBB.1	G	Y	K	H	K	Y		H	K	
BA.4/5	G	Y	K	H	K	Y		H	K	
BQ.1.1	G	Y	K	H	K	Y		H	K	

Supplementary Fig. S1 Key amino acid mutations in the spike protein were identified in Omicron sub-lineages, which were marked in orange.

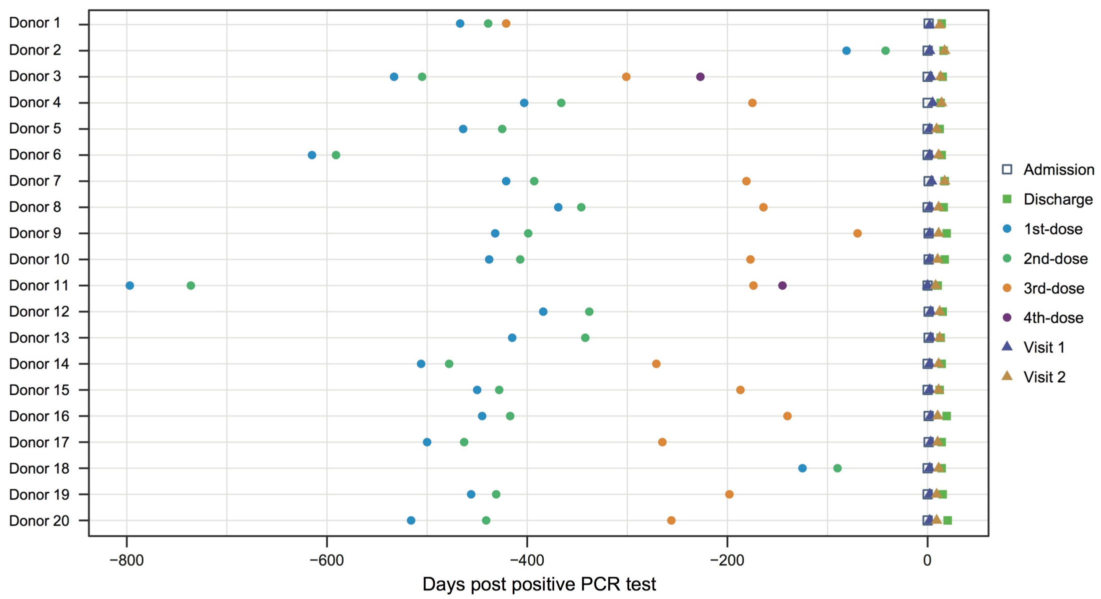

Supplementary Fig. S2 Timeline of events throughout the wild-type (WT) SARS-CoV-2 vaccination, BA.4 or BA.5 breakthrough infection, and follow-up visit.

The information of vaccination, diagnosis, and two follow-up visits were listed. The diagnosis date (PCR test) was normalized to Day 0.

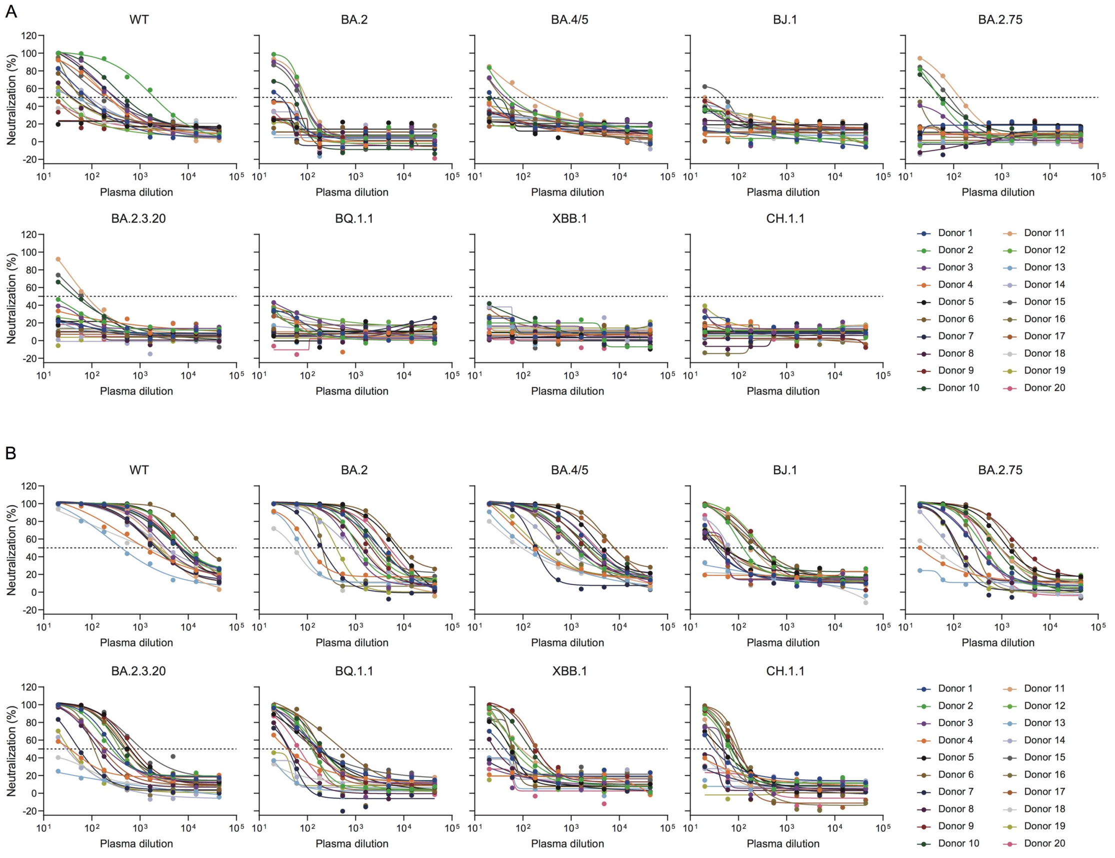

Supplementary Fig. S3 Neutralization curves of plasma samples collected from BA.4 or BA.5 breakthrough infections at Visit 1 (A) and Visit 2 (B) against the WT SARS-CoV-2, BA.2, BA.4/5, BJ.1, BA.2.75, BA.2.3.20, BQ.1.1, XBB.1 and CH.1.1.

One representative curve from two independent experiments was displayed. A cut-off value of 50% in neutralization was indicated by the dotted horizontal line.

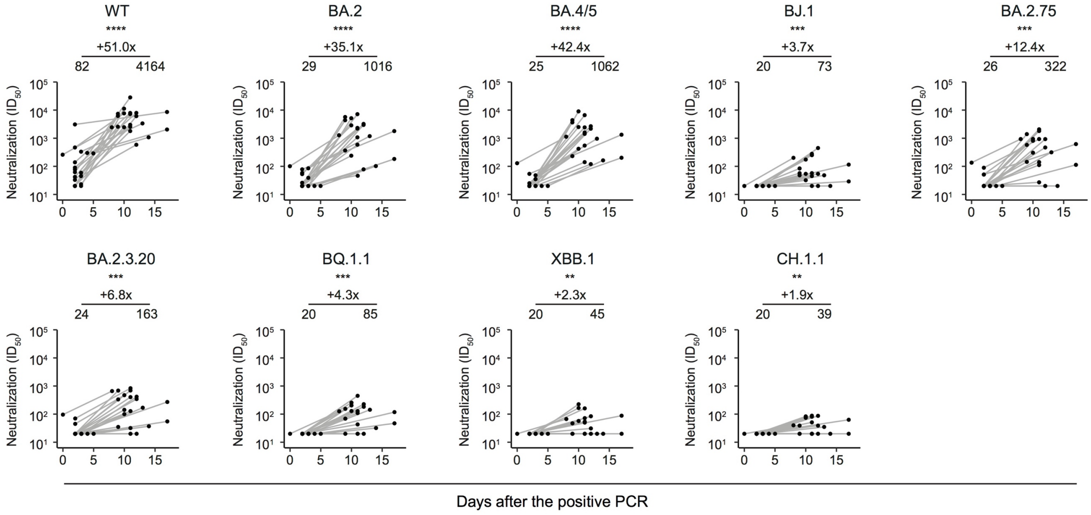

Supplementary Fig. S4 The neutralizing antibody titers of plasma samples collected from Visit 1 (0-5 days after the positive PCR) and Visit 2 (8-17 days after the positive PCR) in 20 individuals.

Geometric mean titer, fold change, and significance of difference are labelled on the top. "+" represents increased neutralization. Statistical significance was performed using two-tailed paired Wilcoxon test. ****, $P < 0.0001$; ***, $P < 0.001$; **, $P < 0.01$.

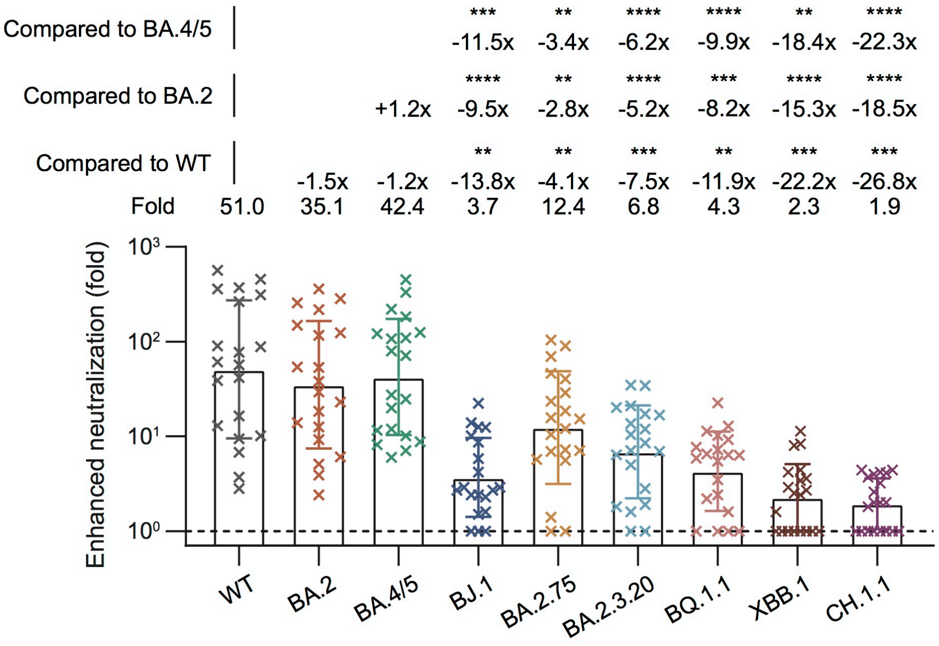

Supplementary Fig. S5 The enhanced neutralization induced by the BA.4 or BA.5 breakthrough infection against WT and a series of Omicron sub-lineages.

Data are presented as geometric mean values ± standard deviation (SD). The fold change and significance of difference are labelled on the top. “-” represents decreased value and “+” represents increased value. The statistical significance was performed using two-tailed Kruskal-Wallis test with paired Wilcoxon's multiple-comparison test. ****, $P < 0.0001$; ***, $P < 0.001$; **, $P < 0.01$.

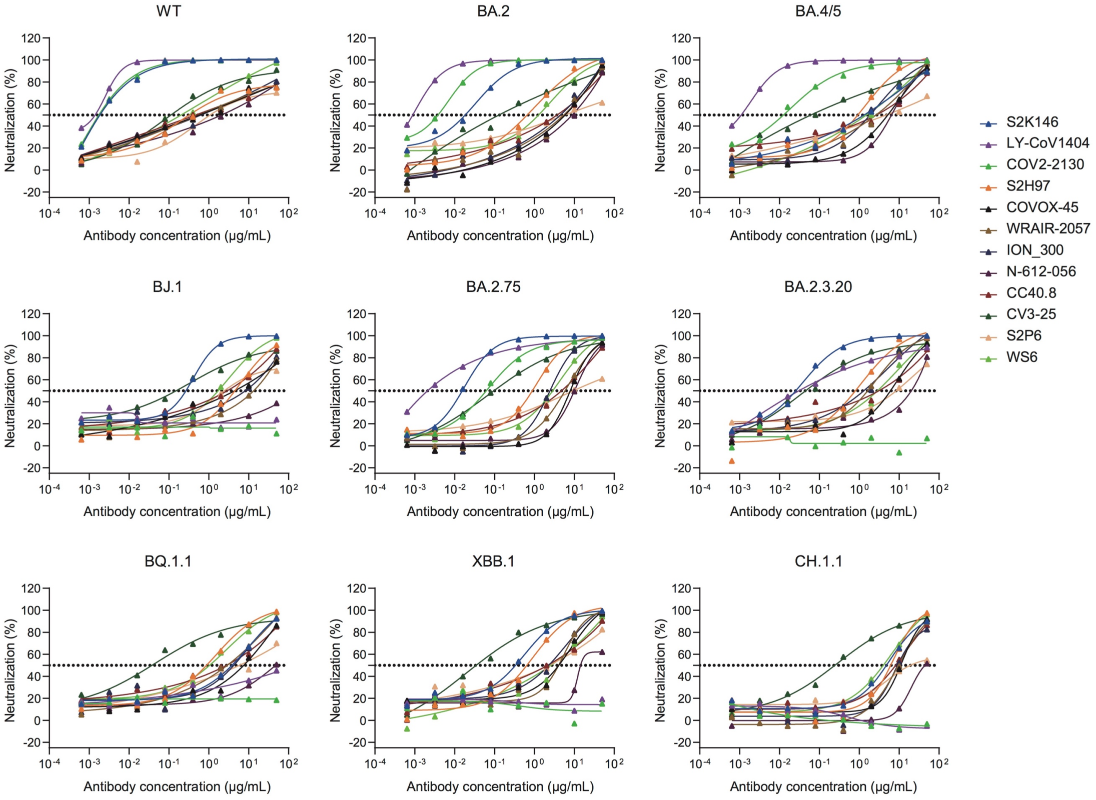

Supplementary Fig. S6 Neutralization curves of 8 RBD-specific and 4 anti-S2 bnAbs against the WT SARS-CoV-2, BA.2, BA.4/5, BJ.1, BA.2.75, BA.2.3.20, BQ.1.1, XBB.1, and CH.1.1.

All bnAbs were serially diluted from 50 μg/mL by 5-fold. One out of two independent experiments was shown. A reduction of 50% in viral infectivity was indicated by a horizontal dashed line.

Supplementary Table S1. Detailed information of subjects involved in this study.

ID	Gender	Age	1st-dose vaccination		2nd-dose vaccination		3rd-dose vaccination		4th-dose vaccination		Positive PCR test	Admission	Discharge	Visit 1	Visit 2	Breakthrough
			Date	Vaccine	Date	Vaccine	Date	Vaccine	Date	Vaccine						
Donor 1	Female	44	2021/4/27	CoronaVac	2021/5/25	CoronaVac	2021/6/12	CoronaVac	/	/	2022/8/7	2022/8/8	2022/8/21	2022/8/9	2022/8/19	BA.4.1
Donor 2	Male	28	2022/5/13	Moderna	2022/6/21	Moderna	/	/	/	/	2022/8/2	2022/8/2	2022/8/18	2022/8/4	2022/8/19	BA.5.2
Donor 3	Female	38	2021/2/19	Sputnik V	2021/3/19	Sputnik V	2021/10/9	Sputnik V	2021/12/22	Sputnik V	2022/8/6	2022/8/6	2022/8/21	2022/8/9	2022/8/19	BA.5.2
Donor 4	Male	52	2021/6/29	BBIBP-CorV	2021/8/5	BBIBP-CorV	2022/2/12	BBIBP-CorV	/	/	2022/8/6	2022/8/6	2022/8/19	2022/8/11	2022/8/20	BA.5.2
Donor 5	Male	35	2021/5/5	CoronaVac	2021/6/13	CoronaVac	/	/	/	/	2022/8/12	2022/8/12	2022/8/24	2022/8/14	2022/8/21	BA.5
Donor 6	Male	16	2020/12/7	CoronaVac	2020/12/31	CoronaVac	/	/	/	/	2022/8/14	2022/8/14	2022/8/28	2022/8/16	2022/8/25	BA.5.2
Donor 7	Female	56	2021/6/21	CoronaVac	2021/7/19	CoronaVac	2022/2/16	CoronaVac	/	/	2022/8/16	2022/8/17	2022/9/2	2022/8/20	2022/9/2	BA.5.6
Donor 8	Male	34	2021/8/14	CoronaVac	2021/9/6	CoronaVac	2022/3/7	CoronaVac	/	/	2022/8/18	2022/8/18	2022/9/3	2022/8/20	2022/8/29	BA.5.2.1
Donor 9	Male	37	2021/6/12	ZF2001	2021/7/15	ZF2001	2022/6/9	ZF2001	/	/	2022/8/18	2022/8/19	2022/9/6	2022/8/20	2022/8/29	BA.5.6
Donor 10	Male	26	2021/6/7	CoronaVac	2021/7/8	CoronaVac	2022/2/23	CoronaVac	/	/	2022/8/19	2022/8/20	2022/9/5	2022/8/21	2022/8/29	BA.5.2.1
Donor 11	Male	34	2020/6/15	BBIBP-CorV	2020/8/15	BBIBP-CorV	2022/2/28	CoronaVac	2022/3/29	CoronaVac	2022/8/21	2022/8/21	2022/8/31	2022/8/21	2022/8/29	BA.5.2
Donor 12	Male	20	2021/8/7	KCONVAC	2021/9/22	KCONVAC	/	/	/	/	2022/8/26	2022/8/27	2022/9/10	2022/8/29	2022/9/7	BA.5.2
Donor 13	Male	27	2021/7/7	KCONVAC	2021/9/18	KCONVAC	/	/	/	/	2022/8/26	2022/8/27	2022/9/8	2022/8/29	2022/9/7	BA.5.2.1
Donor 14	Male	27	2021/4/8	CoronaVac	2021/5/6	CoronaVac	2021/11/29	CoronaVac	/	/	2022/8/27	2022/8/27	2022/9/10	2022/8/29	2022/9/7	BA.5.2
Donor 15	Male	31	2021/6/3	CoronaVac	2021/6/25	CoronaVac	2022/2/21	CoronaVac	/	/	2022/8/27	2022/8/27	2022/9/8	2022/8/29	2022/9/7	BA.5.2.1
Donor 16	Male	36	2021/6/9	KCONVAC	2021/7/7	KCONVAC	2022/4/10	KCONVAC	/	/	2022/8/28	2022/8/29	2022/9/16	2022/8/31	2022/9/7	BA.5.2.1
Donor 17	Female	55	2021/4/15	CoronaVac	2021/5/22	CoronaVac	2021/12/6	CoronaVac	/	/	2022/8/28	2022/8/29	2022/9/11	2022/8/31	2022/9/7	BA.5.2
Donor 18	Male	64	2022/4/26	BBIBP-CorV	2022/5/31	BBIBP-CorV	/	/	/	/	2022/8/29	2022/8/29	2022/9/12	2022/8/31	2022/9/9	BA.5.2
Donor 19	Female	50	2021/5/30	BBIBP-CorV	2021/6/24	BBIBP-CorV	2022/2/12	BBIBP-CorV	/	/	2022/8/29	2022/8/29	2022/9/13	2022/8/31	2022/9/7	BA.5.2
Donor 20	Female	57	2021/3/31	BBIBP-CorV	2021/6/14	CoronaVac	2021/12/16	CoronaVac	/	/	2022/8/29	2022/8/29	2022/9/18	2022/8/31	2022/9/7	BA.5.2

“/”: not available.

Supplementary Table S2. Several key time points during the follow-up visit.

ID	Positive PCR test	Visit 1	Visit 2	Days after the positive PCR		Days between two visits
				Visit 1	Visit 2	
Donor 1	2022/8/7	2022/8/9	2022/8/19	2	12	10
Donor 2	2022/8/2	2022/8/4	2022/8/19	2	17	15
Donor 3	2022/8/6	2022/8/9	2022/8/19	3	13	10
Donor 4	2022/8/6	2022/8/11	2022/8/20	5	14	9
Donor 5	2022/8/12	2022/8/14	2022/8/21	2	9	7
Donor 6	2022/8/14	2022/8/16	2022/8/25	2	11	9
Donor 7	2022/8/16	2022/8/20	2022/9/2	4	17	13
Donor 8	2022/8/18	2022/8/20	2022/8/29	2	11	9
Donor 9	2022/8/18	2022/8/20	2022/8/29	2	11	9
Donor 10	2022/8/19	2022/8/21	2022/8/29	2	10	8
Donor 11	2022/8/21	2022/8/21	2022/8/29	0	8	8
Donor 12	2022/8/26	2022/8/29	2022/9/7	3	12	9
Donor 13	2022/8/26	2022/8/29	2022/9/7	3	12	9
Donor 14	2022/8/27	2022/8/29	2022/9/7	2	11	9
Donor 15	2022/8/27	2022/8/29	2022/9/7	2	11	9
Donor 16	2022/8/28	2022/8/31	2022/9/7	3	10	7
Donor 17	2022/8/28	2022/8/31	2022/9/7	3	10	7
Donor 18	2022/8/29	2022/8/31	2022/9/9	2	11	9
Donor 19	2022/8/29	2022/8/31	2022/9/7	2	9	7
Donor 20	2022/8/29	2022/8/31	2022/9/7	2	9	7
